# Diagnostic Accuracy of Monitoring Tests of Fellow Eyes in Patients with Unilateral Neovascular Age-Related Macular Degeneration: Early Detection of Neovascular Age-Related Macular Degeneration Study

**DOI:** 10.1016/j.ophtha.2021.07.025

**Published:** 2021-12

**Authors:** Sobha Sivaprasad, Katie Banister, Augusto Azuro-Blanco, Beatriz Goulao, Jonathan A. Cook, Ruth Hogg, Graham Scotland, Heinrich Heimann, Andrew Lotery, Faruque Ghanchi, Richard Gale, Geeta Menon, Louise Downey, Nicola Hopkins, Peter Scanlon, Ben Burton, Craig Ramsay, Usha Chakravarthy

**Affiliations:** 1 Moorfields National Institute of Health Research Biomedical Research Centre, London, United Kingdom; 2 Health Services Research Unit, University of Aberdeen, Aberdeen, United Kingdom; 3 Centre for Public Health, Queen’s University Belfast, Belfast, Ireland; 4 Nuffield Department of Population Health, University of Oxford, Oxford, United Kingdom; 5 Health Economics Research Unit, University of Aberdeen, Aberdeen, Scotland; 6 St. Paul’s Eye Unit, Royal Liverpool University Hospitals Foundation Trust, Liverpool, United Kingdom; 7 Faculty of Medicine, University of Southampton, Southampton, United Kingdom; 8 Bradford Teaching Hospitals NHS Foundation Trust, Bradford, United Kingdom; 9 York Teaching Hospital NHS Foundation Trust, York, United Kingdom; 10 Frimley Health NHS Foundation Trust, Surrey, United Kingdom; 11 Hull and East Yorkshire NHS Trust, Hull, United Kingdom; 12 Essex County Hospitals, Colchester, United Kingdom; 13 Gloucestershire Hospitals NHSFT, Cheltenham, United Kingdom; 14 James Paget University Hospital, Gorleston, United Kingdom

**Keywords:** Amsler, Diagnostic accuracy, Fluorescein angiography, Neovascular age-related macular degeneration, OCT, Sensitivity and specificity, Visual acuity, AMD, age-related macular degeneration, CI, confidence interval, DOR, diagnostic odds ratio, EDNA, Early Detection of Neovascular Age-Related Macular Degeneration, FFA, fundus fluorescein angiography, nAMD, neovascular age-related macular degeneration, PHP, preferential hyperacuity perimetry, SD, standard deviation, VA, visual acuity, VEGF, vascular endothelial growth factor

## Abstract

**Purpose:**

To evaluate the diagnostic accuracy of routinely used tests of visual function and retinal morphology compared with fundus fluorescein angiography (FFA) to detect onset of active macular neovascularization in unaffected fellow eyes of patients with unilateral neovascular age-related macular degeneration (nAMD).

**Design:**

Prospective diagnostic accuracy cohort study conducted in 24 eye clinics in the United Kingdom over 3 years.

**Participants:**

Older adults (>50 years) with recently diagnosed unilateral nAMD with a fellow (study) eye free of nAMD.

**Methods:**

Self-reported vision, Amsler, clinic-measured visual acuity (VA), fundus assessment, and spectral domain OCT. The reference standard is FFA.

**Main Outcome Measures:**

Sensitivity and specificity of the 5 index tests.

**Results:**

Of 552 participants monitored for up to 3 years, 145 (26.3%) developed active nAMD in the study eye, of whom 120 had an FFA at detection and constituted the primary analysis cohort. Index test positives at nAMD detection in those confirmed by FFA were self-reported vision much worse (5), distortion on Amsler (33), 10-letter decrease in acuity from baseline (36), fundus examination (64), and OCT (110). Percentage index test sensitivities were: self-reported vision 4.2 (95% confidence interval [CI], 1.6–9.8); Amsler 33.7 (95% CI, 25.1–43.5); VA 30.0 (95% CI, 22.5–38.7); fundus examination 53.8 (95% CI, 44.8–62.5); and OCT 91.7 (95% CI, 85.2–95.6). All 5 index test specificities were high at 97.0 (95% CI, 94.6–98.5), 81.4 (95% CI, 76.4–85.5), 66.3 (95% CI, 61.0–71.1), 97.6 (95% CI, 95.3–98.9), and 87.8 (95% CI, 83.8–90.9), respectively. The combination of OCT with one other index test that was a secondary outcome measure increased sensitivity marginally and decreased specificity for all combinations except fundus examination.

**Conclusions:**

Tests of self-reported change in vision, unmasking of new distortion, measurements of acuity, and fundus checks to diagnose active nAMD performed poorly in contrast to OCT. Our findings support a change to guidelines in clinical practice to monitor for onset of nAMD.

Neovascular age-related macular degeneration (nAMD) is an important cause of severe vision loss in older people because it commonly affects both eyes and has an enormous impact on quality of life.^1^^,^^2^ Over the past decade, treatment with intravitreal anti-vascular endothelial growth factor (VEGF) injections has been shown to be effective in preventing moderate and severe vision loss.^3^ Routine clinical care data obtained from electronic medical records of patients with bilateral nAMD treated with anti-VEGF shows that second eyes with generally good visual acuity (VA) at diagnosis maintain better function over 3 years compared with first eyes that commenced treatment with on average lower levels of VA.^4^ Traditionally, detection of nAMD at onset is made by fundus fluorescein angiography (FFA), which is the confirmatory diagnostic test according to the Age-Related Macular Degeneration Practice Patterns of the American Academy of Ophthalmology.^5^ Fundus fluorescein angiography is an invasive procedure requiring the intravenous injection of fluorescein dye and capture of a sequence of retinal images through a dilated pupil for up to 10 minutes using a specialized camera. This test requires compliant patients, skilled photographers, and medical support for intravenous cannulation and administration of the dye with monitoring for local and systemic reactions including anaphylaxis. The acquired retinal images require review by an experienced retina specialist for the presence of signs of nAMD.

Once nAMD has developed in the first eye, approximately one-quarter of persons will develop neovascularization in their fellow eye within 3 years.^6^ Several strategies are used to detect the onset of nAMD promptly in the unaffected fellow eyes, but these have not been compared to inform what would now be best practice. Clinicians advise their patients to self-monitor the unaffected fellow eye between scheduled clinic visits for symptoms of onset of disease. Patients may be provided with an Amsler chart, which is a high-contrast grid of black lines printed on a white background and is viewed at reading distance monocularly. Visual change such as perceived distortion of the grid can signify the onset of nAMD.^5^^,^^7^ When patients with nAMD in 1 eye attend hospital eye services for treatment, VA, fundus examination, and spectral-domain OCT are commonly used to monitor the fellow eye.^8^ The instrumentation used for OCT is a noncontact, user-friendly noninvasive technology that is used to rapidly acquire depth-resolved images of the posterior fundus allowing the signs of onset of nAMD to be detected.^8^^,^^9^ Although retrospective case series of symptomatic patients imaged with both FFA and OCT show high levels of diagnostic agreement,^10^^,^^11^ our literature searches revealed no robust prospective data and OCT has become adopted into routine clinical practice for establishing a diagnosis of nAMD. One study has suggested a moderate false-positive rate with OCT, increasing the risk of patients being mistakenly diagnosed with nAMD and thus inappropriately treated with costly anti-VEGF therapies.^10^ A newer addition to the noninvasive imaging technologies that can provide information on the ocular circulation is OCT angiography. OCT angiography of the fundus reveals the microcirculation of the retina in exquisite detail that can distinguish vascular components down to the capillaries and resolve the different layers of blood vessels in the retina and choroid. At present, its interpretation, application, and value in determining onset of nAMD remain under investigation.^12^

Because the benefit in terms of maintained good vision is considerably greater if treatment is commenced promptly in confirmed nAMD^4^ and there is a lack of robust evidence of the diagnostic accuracy of commonly used monitoring tests, we conducted a prospective diagnostic accuracy study to evaluate the best test or combination of monitoring tests that can be performed to robustly and efficiently detect nAMD in second eyes of those with nAMD in the first eye.

## Methods

The Early Detection of Neovascular Age-Related Macular Degeneration (EDNA) study was a 3-year multicenter, prospective, cohort, comparative diagnostic accuracy study conducted in a monitoring setting in 24 ophthalmology departments within UK NHS hospitals. The Office for Research Ethics Committees in Northern Ireland reviewed and approved this study (14/NI/1120), and the study was prospectively registered on ISRCTN (48855678). Informed consent was obtained for all patients, and the study followed the Declaration of Helsinki.

Eligibility criteria were persons aged 50 years or more with newly diagnosed nAMD in 1 eye and an unaffected fellow eye confirmed to be free of nAMD by FFA and with a best-corrected VA of ≥ 68 ETDRS letters (Snellen equivalent 20/40 or better) with no confounding retinal pathology. The unaffected fellow eye was the study eye. Exclusion criteria were inability to attend for regular treatment of the first affected eye, refusal of informed consent, and lack of a diagnostic FFA or one that was performed more than 6 weeks before enrollment. The selection criteria were specifically chosen because patients with nAMD attend to receive repeated injections of anti-VEGFs, so enabling regular monitoring of the fellow eyes using a panel of index tests.

### Identification of Participants

Consecutive eligible patients were identified by the clinician or research nurse in the ophthalmology clinics. Potentially eligible patients were invited to attend a baseline study visit where eligibility was assessed in full and written informed consent was obtained.

### Index Tests

We selected 5 tests composed of a mixture of function and morphology that are commonly used and recommended for assessment and monitoring of patients with any stage of age-related macular degeneration (AMD) early or late. These were self-reported vision, the Amsler test, VA, fundus examination, and OCT performed at each visit.

### Self-Reported Vision

Participants were asked a standardized question to ascertain if there had been any subjective worsening of vision in the EDNA study eye compared with the previous clinic visit. These data were collected using a questionnaire.

### Amsler

Participants self-recorded the presence of distortion if any in the EDNA study eye on Amsler charts immediately before or during clinic visits according to instructions that had been provided before enrollment.

### Visual Acuity

All sites recorded the VA from both eyes of participants at every visit. Acuity was measured on EDTRS charts as the number of letters read. Site staff specified whether the acuity assessment was undertaken unaided, with habitual refraction, pinhole, or best corrected with the same method used for baseline and all follow-up visits.

### OCT

OCT images were captured at routine clinic visits using local protocols. Enhanced depth imaging was an optional extra.

### Fundus Examination/Photography

This was assessed by slit-lamp clinical examination or from fundus photography that included fields 1 and 2 comprising the central macula. If a widefield camera was used, a single image was sufficient.

### Participant Follow-up

A fluorescein angiogram conducted at referral confirmed the absence of nAMD in the EDNA study eye. At enrollment, participants were instructed on self-monitoring of vision and testing of the study eye with the Amsler chart. These 2 tests were self-administered before or at each routine clinic visit. All other index tests were conducted during the visit. If the Amsler at the baseline visit was abnormal, participants were eligible to take part in EDNA. However, no further Amsler tests were collected during follow-up visits. The protocol required the patient to undergo an FFA in the event that any of the index tests were positive (a trigger) for nAMD. In the event of a positive FFA, the participant exited the study. All participants with negative FFA were followed up for a minimum of 30 months or up to 3 years. In the absence of a trigger arising from a positive index test, 2 additional planned study visits were undertaken at month 18 and on completion of the study (study exit). At these visits, protocol-based study procedures for the measurement of all index tests, self-reported vision, Amsler, VA retinal imaging (color photography, SD-OCT), and fluorescein angiography were performed (Fig S1, available at www.aaojournal.org).

## Definitions of Positive Index Tests

1. Self-reported vision: A positive test was defined as a worsening reported by the participant for subjective assessment of vision in the EDNA study eye. This was assessed using the following question: “How is your vision in the (unaffected) eye compared to the last visit?” with the following 4 possible answers: “about the same or better,” “a bit worse,” “worse,” or “much worse.” “Much worse” triggered an FFA.
2. The Amsler test: A positive Amsler test was defined as the reporting by the patient of the appearance of distortion in the grid or disappearance of regions of the grid pattern (scotoma) on the Amsler chart. Because of the known high rate of false-positives, this test was only performed during follow-up after confirming the existence of a distortion free Amsler test completed at baseline.
3. Visual acuity tested on ETDRS vision charts: A positive test was defined as a reduction of 10 or more letters in VA from the baseline measurement.
4. Fundus clinical evaluation: Biomicroscopic examination of the eye or interpretation of the color fundus images was made by an appropriately qualified member of the study team. A positive test was the presence of clinical signs of active nAMD on slit-lamp biomicroscopy that included elevation of the macular retina or the presence of edema, hemorrhage, lipid exudates, or detection of these features on the color image.
5. OCT: The interpretation of OCT scans at each visit was made by the site clinician. A positive test was defined as the presence of signs of clinically relevant and active nAMD on constituent B scans of the macular raster captured by spectral domain OCT. Clinicians classified OCT as exhibiting nAMD if there were regions of hyporeflectivity within the neurosensory retina indicating accumulation of intraretinal fluid between the neurosensory retina and the retinal pigment epithelium representing subretinal fluid or an increasing pigment epithelial detachment. Shallow irregular elevation of the retinal pigment epithelium without subretinal fluid was not considered a trigger. Regions of hyperreflectivity in the subretinal space (subretinal hyperreflective material) indicating the presence of abnormal blood vessels, fibrin, and cellular elements were a trigger.

### Reference Standards

The findings of the FFA at capture in the clinic were the primary reference standard read by the site clinician who was not masked to index test findings. The classification of the FFA was based on traditional angiographic interpretation of the leakage patterns on FFA leading to the diagnosis of the presence of nAMD.

We prespecified 2 additional reference standards (Table S1, available at www.aaojournal.org). An enhanced reference standard that was an independent assessment of the FFA by an ophthalmic reading center was included because it is known that disagreements can exist between clinician detection of the onset of nAMD on an FFA and that undertaken by trained graders.^13^ Graders were masked to index test results and evaluated the FFA for features of nAMD based on a prespecified grading protocol. A pragmatic reference standard included clinician decision on conversion to nAMD based on FFA and those who could not be imaged with FFA at the time of onset of nAMD because of health concerns or because the participant declined. The FFA procedure and its interpretation in determining the primary and enhanced reference standards are described in Appendix S1 (available at www.aaojournal.org).

## Outcomes

### Primary Outcome

The primary outcome was the sensitivity and specificity of the index tests compared with the primary reference standard and only included participants whose diagnosis of conversion to nAMD or not in the EDNA study eye was based on the FFA interpretation by the site clinician.

### Secondary Outcomes

Secondary outcomes included sensitivity and specificity of the index tests compared with the enhanced reference standard, diagnostic odds ratio (DOR), likelihood ratio, and proportions of indeterminate tests. Other outcomes included the sensitivity and specificity of combinations of tests.

## Sample Size

The sample size calculation was based on comparative diagnostic accuracy to ensure the ability to detect differences in sensitivity and specificity between candidate tests based on McNemar’s test.^14^ Under the primary analysis, a positive candidate test result was defined as any positive result during the monitoring period on the respective test. At 2-sided 5% significance level and 90% power, a paired difference of 15% (80% to 65%) in sensitivity required 491 participants (560 allowing for indeterminate/missing data results, including participants lost to follow-up cumulatively of up to 12%) given a cumulative incidence of 28% at 3 years.^15^ A smaller difference in specificity was also identifiable (7%; 94% to 87% with power and significance levels as before). The sample size was also sufficient to detect differences in sensitivity and specificity of 20% or more at the same power and significance levels even if the sensitivities/specificities were substantially lower (e.g., 60% to 40%) or the previous difference if the level of missing data is higher (e.g., 20%). These calculations assumed disagreement between tests was at least at the level observed in a similar analysis of data on another eye condition (74% of maximum possible; disagreement of 0.30 and 0.13 for the main sensitivity and specificity calculations).^16^

### Statistical Analyses

We conducted our analyses using Stata version 15 (StataCorp LP) following a predefined statistical analysis plan. We summarized the baseline characteristics of participants. If no reference standard was available at any point after baseline, the participant was excluded from all subsequent analyses.

For each participant, we defined the end of the follow-up as the date of the last FFA providing 1 of the following 2 criteria were met: (1) The final FFA showed no nAMD and there was at least 1 prior index test result; (2) the final FFA showed nAMD and an index test result was obtained within the previous 3 months. If neither criterion was met, we used the result of an FFA from a prior visit to define the end of the monitoring period.

We then collapsed the test results obtained from baseline until the end of the valid follow-up period, defined earlier, to obtain a single positive or negative test result. We classified any positive test over the valid follow-up period as an overall positive result. To be a negative test result, all test results over the valid follow-up period had to be negative. Cross tabulations of index tests against the primary reference standard were performed.

We had multiple reference standards, but our prespecified primary reference standard was clinician determination of conversion to nAMD based on the FFA. For all reference standards, we calculated sensitivity and specificity with 95% confidence intervals (CIs) using the Agresti-Coull method and positive and negative likelihood ratios with 95% CIs using the method proposed by Zhou et al.^17^ We also computed likelihood ratios, which are an estimate of the probability of accuracy of detection of disease by the index test, considered by some to be the preferable means of summarizing of diagnostic accuracy of a test. We calculated the DORs and the proportion of indeterminate tests with 95% CIs. We compared the monitoring sensitivity and specificity of the tests using McNemar’s statistical test (with 95% CIs produced using Newcombe’s method).^18^ Additional analyses assessed the sensitivity and specificity of the index tests under the following conditions: varying the definition of the time period over which a positive index test was considered (the last 6 months of participants’ follow-up or using only the last available index test). When cases were included in the analysis, we handled missing data in several ways. If according to the protocol a reference standard should have been performed but was not undertaken, the missing reference standard was presumed to indicate a negative result at that visit. When FFAs were deemed to be indeterminate, they were treated as missing, and sensitivity analyses were performed to assess the impact of this assumption. Similar analyses were conducted using the enhanced reference standard.

By using the primary reference standard and all available index test results over the full monitoring period, the effect of combinations of OCT with each of the other index tests was investigated. The positive result was defined in 2 ways: (1) when either of the tests was positive and (2) when both tests had to be positive. We also examined 2 additional combinations of index tests after excluding OCT: (1) the combination of the 4 index tests other than OCT and (2) the combination of only those index tests that assessed function (self-reported vision, Amsler test, and VA).

## Results

### Participant Characteristics

**Figure 1:**
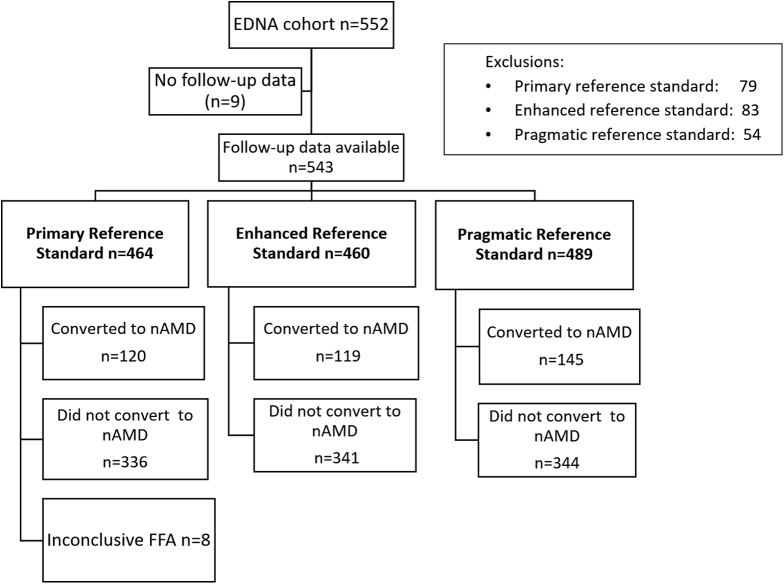
Flowchart of participants included in the main and enhanced reference standards. EDNA = Early Detection of Neovascular Age-Related Macular Degeneration; FFA = fundus fluorescein angiography; nAMD = neovascular age-related macular degeneration.

Between June 2015 and March 2017, 949 potential participants seen in 24 NHS eye clinics were approached, of whom 578 consented to take part in the EDNA study. Figure 1 shows the flow of participants from enrollment until exit or development of nAMD in the EDNA study eye. After excluding those who had been consented in error or withdrew consent, 552 formed the final cohort, and the baseline characteristics of this group are shown in Table 1.

**Table 1:** Participant Characteristics (n = 552)

Characteristic	n (% n/552)
Age, yrs, mean (SD), n	77.4 (7.7), 552
BMI - mean (SD), n	27.6 (5.3), 423
Male gender	236 (42.8)
Hypertension∗	292 (52.9)
Cardiovascular disease∗	118 (21.4)
Family history of AMD∗	82 (14.9)
History of diabetes∗	88 (15.9)
Other systemic disorders∗	48 (8.7)
Nutritional supplements∗	165 (29.9)
Current smoker∗	70 (12.7)
Ex-smoker	264 (47.8)
Never smoked	217 (39.3)
**Ocular characteristics in the EDNA study eye**	
Study eye is better-seeing eye	525 (95.1)
VA (ETDRS letters) in study eye (mean, SD)	79 (5.4)
Amsler test scotoma present†	92 (16.7)
Scotoma absent	460 (83.3)
**Lens status**^†^	
Cataract present	285 (51.6)
Phakic cataract absent	148 (26.8)
Pseudophakic	117 (21.2)

BMI = body mass index; EDNA = Early Detection of Neovascular Age-Related Macular Degeneration; VA = visual acuity.

∗ Clinical history was not captured in 1 participant.

† Amsler test and lens status missing in 2 participants.

The average number of clinic visits for the entire cohort was 15.6 (standard deviation [SD], 7.7) during follow-up (range, 1–35). In participants who developed nAMD in the study eye, the average number of clinic visits was 10.1 (SD, 6.7) and 19.0 (SD, 6.0) for those who did not develop nAMD. The number of visits/tests completed per participant over the 3-year period is shown in Table S2 (available at www.aaojournal.org). The proportion of visits with index tests completed were self-reported vision 90%, Amsler 88%, VA check 99%, fundus examination 91%, and OCT 93%. The average follow-up period in participants who developed nAMD in the study eye was shorter at 14 months (IQR, 7.7–23.0) compared with those who did not develop nAMD, which was 35 months (IQR, 29.7–36.5). A positive Amsler test was recorded in 92 participants (17%) at the baseline visit, so this index test was not conducted during follow-up in this subset. During the study, 145 participants developed active nAMD in the EDNA study eye. In 120 participants (83.0%), a confirmatory FFA was available and read by the site clinician. The crude conversion rate of development of new nAMD (based on the 456 participants who constituted the primary reference standard) in the EDNA study eye was 26% (95% CI, 22–31) with a median follow-up time of 33 months (range, 0.8–38.5 months) (Fig S2, available at www.aaojournal.org).

Table S3 (available at www.aaojournal.org) shows the interval in days between the date of the primary reference standard and that of the relevant index test grouped by conversion to nAMD in the study eye. In the majority of participants who converted to nAMD, the positive index test triggered the reference standard on the same day. In participants who did not develop nAMD in their study eye during follow-up, the interval between final set of index tests and the final FFA ranged from an average of 8 days for self-reported vision and 117 days for VA.

### Diagnostic Accuracy

The numbers of participants included in the primary, enhanced, and pragmatic reference standards are shown in Figure 1 and Table S4 (available at www.aaojournal.org). Table 2 shows cross tabulations of index test results versus the primary reference standard. In participants with FFA-confirmed new-onset nAMD with data on subjective vision loss in the EDNA study eye (n = 118), self-reported vision was “a bit worse” in 19, “worse” in 13, and “much worse” in 5. The Amsler was positive in 33 (n = 98). Reduction in VA in the EDNA study eye from baseline to onset of nAMD of 10 or more letters was recorded in 36 (n = 120), whereas a decrease of 5 or more letters and 15 or more letters was recorded in 77 and 22 participants, respectively. Fundus examination/color imaging revealed new nAMD in 64 (n = 119). Features of onset of nAMD were recorded as present on OCT in 110 of the 120 confirmed conversions.

**Table 2:** Cross Tabulations of Index Test Results versus Primary Reference Standard (n = 456)

Index test	FFA +VE	FFA -ve	All
Self-reported vision same	81	280	361
Self-reported vision bit worse	19	30	49
Self-reported vision worse	13	18	31
Self-reported vision much worse∗	5	8	13
All	118	336	454
Amsler +ve	33	52	85
Amsler -ve	65	227	292
All	98	279	377
VA fall of 5 letters +ve	77	231	308
VA fall of 5 letters -ve	43	104	147
VA fall of 10 letters +ve∗†	36	113	149
VA fall of 10 letters -ve†	84	222	306
VA fall of 15 letters +ve	22	55	77
VA fall of 15 letters -ve	98	280	378
VA fall of 20 letters +ve	13	6	77
VA fall of 20 letters -ve	107	329	378
All	120	335	455
Fundus clinical exam +ve	64	8	72
Fundus clinical exam -ve	55	327	382
All	119	335	460
OCT +ve	110	41	151
OCT -ve	10	294	394
All	120	335	455

FFA = fundus fluorescein angiography; VA = visual acuity.

+ve refers to a positive test as defined in the prespecified statistical analysis plan. For self-reported VA, a positive test result was much worse. A positive Amsler is one for which distortion or blank spaces were reported where previously there was none. Decrease in VA of 10 or more letters was considered a positive test result. Fundus clinical examination was positive if signs of new-onset nAMD was detected on slit-lamp biomicroscopy or a color photograph. OCT was test positive if new-onset intraretinal or subretinal fluid or subretinal hyperreflective material was detected. The -ve indicates the numbers in which the index tests were classified as negative.

Missing data. A follow-up FFA was not acquired in 79 participants, of whom 25 were clinically judged to have developed nAMD. The FFA was inconclusive in 8 participants. Participants who did not have a follow-up FFA or in whom the FFA was inconclusive were excluded from the primary reference standard. Amsler was not performed in 90 participants who had a positive Amsler at baseline. At follow-up, Amsler and self-reported VA were missing in 2 participants. Clinic VA was missing in 1 participant.

∗ An FFA was triggered by a positive test.

† The prespecified primary analysis was based on a decrease of 10 letters from baseline at any time point during the follow-up period.

When FFA was negative for new nAMD, 10 of 337 self-reported “much worse” vision loss, 52 of 279 had a positive Amsler test, and 113 of 335 had a 10-letter VA loss recorded by that visit. Features of onset of nAMD were noted as present on fundus clinical examination in 8 of the 335 in whom FFA confirmed the absence of neovascularization. Clinical signs of exudation were deemed as present in the OCT scans of 41 of the 335 with a negative FFA (Table 2).

**Figure 2:**
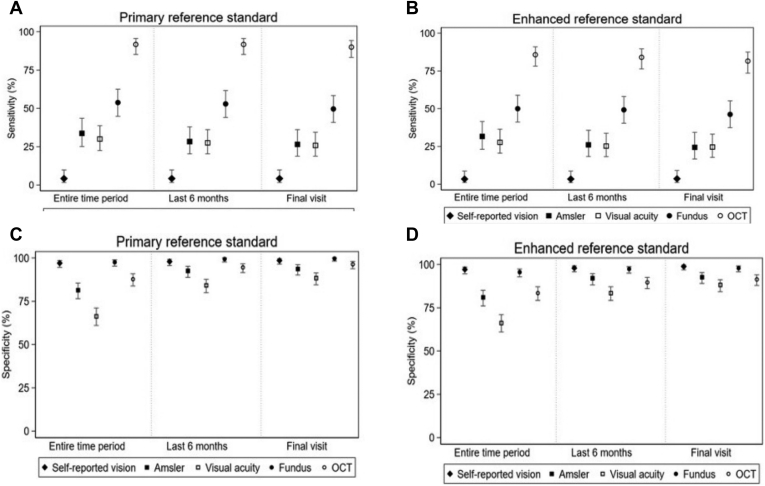
Sensitivity of index tests **(A)** using the primary reference standard (clinician determination based on FFA) and **(B)** using the enhanced reference standard (reading center determination based on FFA). Test performance is shown in the 3 panels separated by **dotted lines**. The time periods are all visits, last 6 months of follow-up, and final visit. Index test performance remained similar regardless of the time period, with sensitivities that are lowest for self-reported vision moderate and similar for Amsler test and visual acuity (VA), good for fundus examination, and best for OCT. Specificity of index tests **(C)** using the primary reference standard (clinician determination based on FFA) and **(D)** enhanced reference standard (reading center determination based on FFA). The time periods are all visits, last 6 months of follow-up, and final visit. Index test performance. Specificities were clustered at the high end and the performance of the index tests were not altered by changing the time period. EDNA **=** Early Detection of Neovascular Age-Related Macular Degeneration; FFA = fundus fluorescein angiography; nAMD = neovascular age-related macular degeneration.

Sensitivity and specificity of the index tests were calculated on the basis of the primary reference standard of FFA read by the site clinician and determined to have developed active nAMD. Table 3 shows that the sensitivity of OCT was markedly higher at 91.7% compared with those of all other tests that ranged from 4.2% to 53.8%. Fundus clinical examination had the highest specificity (97.9%), followed by self-reported vision (97%) and OCT (87.8%). Changing to the enhanced reference standard (Table 4) resulted in a small reduction of sensitivity and specificity of the OCT compared with the primary reference standard. Specificities remained largely unchanged for all the index tests between enhanced and primary reference standards. Table S4 (available at www.aaojournal.org) shows the cross tabulations of clinician determination of conversions to nAMD versus the reading center. Figure 2A to D summarizes the sensitivities and specificities of the index tests against the primary and enhanced reference standards and for the secondary analyses varying the definitions of the time periods over which the index tests were considered. Table S5 (available at www.aaojournal.org) shows the sensitivity and specificity of the index tests against clinical judgment of conversion to active nAMD in the 145 participants. Pairwise comparisons of sensitivity and specificity of the various index tests revealed statistically significant differences **(**Table S6, available at www.aaojournal.org).

**Table 3:** Sensitivity and Specificity of Index Tests Against the Primary Reference Standard (Clinician Determination of Conversion to nAMD Based on FFA)∗

Index Test	Primary Reference Standard			
	Sensitivity (%) (95% CI)	True-Positives/Participants with nAMD	Specificity (%) (95% CI)	True-Negatives/Participants without nAMD
Self-reported vision†	4.2 (1.6–9.8)	5/118	97.0 (94.6–98.5)	327/337
Amsler test‡	33.7 (25.1–43.5)	33/98	81.4 (76.4–85.5)	227/279
VA§	30.0 (22.5–38.7)	36/120	66.3 (61.0–71.1)	222/335
Fundus clinical examination‖	53.8 (44.8–62.5)	64/119	97.6 (95.3–98.9)	327/335
OCT¶	91.7 (85.2–95.6)	110/120	87.8 (83.8–90.9)	294/335

CI = confidence interval; FFA = fundus fluorescein angiography; nAMD = neovascular age-related macular degeneration.

∗ A total of 464 participants were included in the analysis with 78 excluded because of missing data; 120 study eyes developed nAMD, 337 did not, and 8 had an inconclusive FFA.

† 1 missing;

‡ 2 missing;

§ 1 missing;

‖ 2 missing;

¶ 1 missing.

**Table 4:** Sensitivity and Specificity of Index Tests Against the Enhanced Reference Standard (Reading Center Determination of Conversion Based on FFA)

	Sensitivity (%) (95% CI)	True-Positives/Participants with nAMD	Specificity (%) (95% CI)	True-Negatives/Participants without nAMD
Self-reported vision∗	3.4 (1.1–8.7)	4/117	97.1 (94.6–98.5)	331/341
Amsler test†	31.6 (23.1–41.5)	30/95	81.0 (76.0–85.1)	230/284
VA‡	27.7 (20.5–36.4)	33/119	66.2 (61.0–71.0)	225/340
Fundus clinical examination§	50.0 (41.1–58.9)	59/118	95.6 (92.8–97.4)	325/340
OCT‖	85.7 (78.2–91.0)	102/119	83.5 (79.2–87.1)	284/340

CI = confidence interval; FFA = fundus fluorescein angiography; nAMD = neovascular age-related macular degeneration; VA = visual acuity.

A total of 460 participants were included in the analysis, and 83 were excluded because of missing data; 119 study eyes developed nAMD, and 341 did not.

∗ 2 missing;

† 2 missing;

‡ 2 missing;

§ 1 missing;

‖ 1 missing.

The positive and negative likelihood ratios (and 95% CI) are shown in Table S7 (available at www.aaojournal.org). OCT had a high positive likelihood ratio and very low negative likelihood ratio. Fundus clinical examination had a high positive likelihood ratio, but the negative likelihood ratio was fivefold greater than for OCT. Estimated DORs varied greatly from 0.8 (VA) to 79.5 (OCT).

### Sensitivity and Specificity of Combinations of Tests

On examining the combination of OCT and 1 other index test when either test could be positive for an overall positive result, sensitivity increased marginally for combinations, achieving 96% for OCT and VA, and specificity decreased except for OCT combined with fundus examination (Table S8, available at www.aaojournal.org). When both tests had to be positive (Table S9, available at www.aaojournal.org), for the combination to be classified as overall positive, sensitivity was reduced for all combinations compared with that of OCT by itself but was offset by improvements in specificity (although of smaller magnitude). By combining all index tests except OCT, the sensitivity of this combination was lower than that achieved with OCT alone. By excluding fundus clinical examination, with the combinations consisting only of tests that reflected function (self-reported vision, Amsler test, and VA), sensitivity decreased by approximately 20%. For combinations that excluded OCT or both OCT and fundus clinical examination, specificities were similar and remained lower than combinations that included OCT (Table S10, available at www.aaojournal.org).

## Discussion

This diagnostic accuracy study of monitoring fellow eyes of participants with unilateral nAMD at baseline compared 5 commonly performed index tests with the reference standard of FFA. On the basis of the detection of conversion to nAMD in the study eye by FFA, we have shown conclusively that compared with the other index tests, OCT has the best diagnostic accuracy (highest combination of sensitivity and specificity) with clear statistical evidence of differences in performance independent of the monitoring periods chosen for at least up to 3 years after the diagnosis of nAMD in the first eye. We varied the reference standard and index test definitions to mimic clinical and research situations and considered combinations of index tests. We found that all analyses pointed clearly to OCT as the most accurate index test and suitable for use on its own. Although self-reported vision and clinical examination showed high specificity, these and the other index tests apart from OCT performed poorly for sensitivity and are therefore unsuitable for monitoring for the onset of nAMD. In addition to the diagnostic accuracy of an index test, we took into consideration the ease of performing a test and its availability for monitoring in routine clinical practice in the current UK NHS settings. We showed that the index tests were conducted at a very high proportion of visits. Because data were collected during usual care, the findings from this pragmatic study are readily applicable in routine clinical practice.

Data are limited on the accuracy of monitoring tests of fellow eyes of patients with nAMD in 1 eye. In a cohort study on 98 individuals with unilateral nAMD, Do et al^19^ monitored the unaffected fellow eyes with OCT, supervised Amsler test, preferential hyperacuity perimetry (PHP), and best-corrected VA every 3 months for 2 years or until development of nAMD for 2 years, which occurred in 17%. The tests showed low to moderate sensitivity (≤0.5), but this is unsurprising, because the time domain OCT used by Do et al^19^ has poor vertical resolution. The PHP test is performed on specialized equipment and requires patient training and compliance, with a high proportion failing to perform the test properly, which made it unsuitable for routine use.^20^ The Home study, which was nested within the Age-Related Eye Diseases Study 2, reported that the ForeseeHome, a device based on PHP designed for use in the community, had a sensitivity of 82% and specificity of 88% compared with a sensitivity of 70% and specificity of 95%, which was based on detection of new-onset nAMD on color stereo photographs read by a group of retina specialists.^21^ The Home study compared the ForseeHome system with self-monitoring of visual symptoms in the detection of incident nAMD, and the mean VA loss at the time of detection of new nAMD in the former was 4 letters compared with 9 letters in the latter.^21^ Self-reported decline in visual function and standard nonrefracted clinic-based VA, clinical examination, and OCT were not included; therefore, the data from the Home study are not directly comparable to the findings from EDNA. A recent analysis of the ForeseeHome device reported that its utility in the detection of new-onset nAMD based on data collected from routine clinical care fell considerably short of its previously reported accuracy.^21^ Notably, a small proportion of the sample were able to establish a baseline, and of those who did, 92% of the alerts were false-positive. The foregoing emphasizes the need for continuing evaluation of monitoring devices that can be used in the home setting before their introduction into routine clinical practice for the detection of the onset of nAMD. In this context, recent developments in devices that are capable of monitoring of patients with exudative maculopathies with OCT in the community also require rigorous evaluation.

With respect to other studies that have compared OCT with FFA, one that was undertaken in the clinical setting on patients referred for nAMD reported a sensitivity and specificity for OCT alone in detecting nAMD as 100% and 80.8%, respectively.^10^ However, 17% were false-positives. Furthermore, this study differs from EDNA in its cross-sectional design, retrospective analysis, and a lack of any other commonly performed monitoring tests.^10^

An important finding of some concern in EDNA was the poor diagnostic accuracy of self-reported visual function. Less than one-fifth of the participants reported a change in vision that was worse or much worse at detection of onset of nAMD in the EDNA study eye. Additionally, approximately 10% of our patients had worsening of vision that was shown not to be due to the onset of nAMD. Our study monitoring committee included a number of patient experts who were involved in the study design and interpretation of findings. One reason suggested by one of our patient experts was that any sudden deterioration in acuity that occurs at onset of nAMD had been masked by a gradual decline in vision in the interim, which in itself may have triggered adaptive strategies. The finding that it is not possible to rely on self-reported visual deterioration as a marker for the onset of nAMD is particularly disturbing because the study eye was required to have a VA of 68 or more ETDRS letters (Snellen 20/40 or better) at enrollment. This degree of function represents driving-level VA, and it is notable that in more than 95% of participants the study eye was the eye with better acuity. Thus, the inability of our patient population to recognize a change in function in the EDNA study eye around the time of onset of nAMD is both surprising and worrying because it is common clinical practice to ask patients to self-monitor for worsening of vision or the onset of distortion.^5^ The other self-administered test, which involves recording the appearance of a scotoma or a distortion on the Amsler chart, also had poor sensitivity and specificity, which is a recognized drawback of this tool.^20^

It was notable that a decrease in VA measured at clinic visits also showed poor sensitivity. We considered a decrease of 10 letters (2 ETDRS lines) as an event that would be recognized by participants as a change in function of sufficient severity that could be due to the onset of nAMD. However, among the 120 participants who were confirmed with new-onset nAMD in the study eye, less than half had a decrease in VA of 10 or more letters, and conversely of the 335 who did not develop nAMD, around one-third had a decrease in VA of ≥ 10 letters. Possible reasons, including VA loss secondary to atrophy of the macular tissues^22^ and cataract,^23^ emphasize the need for awareness of comorbidities that can result in functional impairment in this age group. Furthermore, inter-session repeatability of VA measurements can vary considerably. The poor performance of VA is a major concern for several reasons. First, because of the COVID crisis, there is currently a strong emphasis on home monitoring for detection of new-onset nAMD, which include web-based applications of the Amsler test and visual function measurements. Our study clearly confirms that reliance on psychophysical technologies that measure visual change or self-reported symptoms will result in both a lower detection rate and higher false-positive rate. Considering this risk-benefit ratio, it is important to evaluate home-monitoring devices using visual function to monitor AMD diagnosis or treatment response in pragmatic trials conducted in real-world settings before introducing them into clinical practice.^21^^,^^24^ Second, to minimize patient interaction, many clinics are currently choosing not to perform a monitoring OCT in patients who attend for follow-up treatment in their first eye. Our data show that asking patients to report the appearance of new symptoms or reliance on measures of acuity, even those obtained in the clinic, can result in underdetection of the onset of nAMD in the at-risk eye. In fact, our study results recommend further research on remote monitoring using OCT rather than visual function measures.^25^

### Study Strengths and Limitations

Our study has multiple strengths. First, EDNA is the largest prospectively designed multicenter monitoring study of patients with existing nAMD in 1 eye that compared multiple pragmatic candidate tests in their ability to detect the onset of nAMD in the contralateral eye, which was the designated study eye. In addition to sensitivity and specificity, with the standard measures of diagnostic accuracy, following our prespecified statistical analysis plan, we calculated likelihood ratios and DORs providing further clarity on the performance of the index tests. Likelihood ratios indicate the clinical implications of using a test more clearly than sensitivity and specificity. Tests may be better at ruling in disease (higher positive likelihood ratio) or ruling out disease (lower negative likelihood ratio). The DOR reflects a single combined quantification of the diagnostic value of the test. OCT had the highest positive likelihood ratio and lowest negative likelihood ratio, thus indicating it performed better than any of the other tests in terms of both ruling on the onset of nAMD and ruling out absence of conversion to onset of nAMD. Second, EDNA is representative of a wide population because patients were recruited from 24 NHS Trusts across the United Kingdom and the rate of progression to nAMD in the EDNA study eye was comparable to previous longitudinal cohorts with nAMD in the contralateral eye,^6^^,^^15^ emphasizing the generalizability of our study results. Third, index tests were captured at most routine clinic visits with only a small proportion not carried out when a participant had attended a visit. Fourth, through inclusion of FFA at month 18 and the exit visit, we were confident that study eyes were free of nAMD at these time points. This allowed us to obtain robust estimates of sensitivity and specificity of the index tests under the principal analysis. Fifth, because clinicians do not agree in the interpretation of an FFA^26^ and disagreements can also exist between clinician detection of the onset of nAMD and trained graders working in a reading center,^13^ we included an enhanced reference standard. Although the primary reference standard representing routine clinical practice may be associated with high risk of bias, the enhanced reference standard that was provided by masked graders in an independent reading center confirms the validity of our findings. We included persons with AMD as advisors in our study steering group, who helped in the conduct and interpretation of the study findings.

Our study has several limitations. First, we did not include OCT angiography as one of the test technologies. This is a recently introduced, novel, noninvasive method of imaging the retinal and choroidal vasculature with potential to reveal the presence of neovascular complexes of nAMD.^27^ Although OCT angiography has become rapidly adopted within the retina community in the developed world as a confirmatory test for nAMD, it is not yet widely available in many countries. Furthermore, reports on its accuracy compared with FFA suggest that diagnosis may be confounded by artifacts and other conditions.^12^^,^^28^^,^^29^ Second, in 12% of participants who were diagnosed as having developed nAMD during follow-up, FFA was lacking. In most cases, this was due to patient preference not to have the invasive test or clinical caution in persons who had developed mild allergic reactions to previous injections of fluorescein dye. However, sensitivity analyses based on the clinical diagnosis of conversion to nAMD did not alter the findings (data shown in supplementary materials, Table S4 and S5). Third, 25 participants (representing 17% of the 145 who were classified as having developed active nAMD) did not have a confirmatory FFA and were excluded from the calculations of diagnostic accuracy in the main analysis. Nonetheless, because the main analysis showed that the sensitivity of OCT was extremely high at 92%, it is highly likely that if the remaining 25 participants had been imaged with FFA, a similar performance would have been observed. We note the similarity in test performance between the primary and pragmatic reference standards. Fourth, our study enrolled persons who had a confirmed diagnosis of nAMD in 1 eye already and therefore had a high probability that macular pathology that developed subsequently in the fellow eye was likely due to AMD. We determined the sample size and selection criteria based on the most robust data for both rate and highest risk of conversion to nAMD.^30^ Our study did not include persons with bilateral intermediate AMD, who are also at increased risk,^30^ but the reported rates of conversion in such a group would have necessitated a large sample, which was not possible within the allocated resources. We recognize that other exudative maculopathies that also occur in the older age groups can exhibit similar features on OCT and may result in misclassification and a higher false-positive rate. Nonetheless, our data offer strong support for the use of OCT for the detection of nAMD at earliest onset in the wider population. Fifth, in a small proportion of participants who were classified as not having converted to nAMD in the EDNA study eye, the FFA reference standard was not carried out for repeatedly positive index tests because of its invasive nature and concerns about patient safety. This approach reflects clinical practice in the United Kingdom, but because additional FFAs at 18 and 36 months in the study were undertaken, it is unlikely that any true conversions were missed. Last, as participants exited the study at onset of nAMD in the study eye and were treated, we were unable to assess whether tests with a false-negative result might have become positive at a later stage. Despite these limitations of our study, the diagnostic accuracy findings in this monitoring context are striking and robust.

In conclusion, the findings of the EDNA study are of relevance globally because the monitoring strategy outlined in currently applicable guidelines, such as the American Academy of Ophthalmology Preferred Practice Pattern,^5^ places high reliance on self-reporting of change in visual function and Amsler test, which has been shown in EDNA to have poor sensitivity and only moderate specificity. Because the risk of progression to nAMD in the unaffected fellow eyes of a patient with nAMD in 1 eye may be as high as 50% within 5 years of diagnosis,^6^ the use of OCT as a monitoring tool has considerable potential for cost savings.^9^^,^^31^ Treatment for nAMD imposes a significant burden for patients, their carers, and hospital facilities, and a diagnostic test of such high sensitivity and specificity has important implications in terms of acceptability to patients and can help streamline care pathways and reduce the impact on hospital intravitreal injection services. In terms of diagnostic accuracy, based on our findings presented, a revised strategy to monitor such patients with OCT should be considered.
