# Corrigendum

**DOI:** 10.1016/j.ophtha.2025.03.015

**Published:** 2025-06

The authors of “Diagnostic accuracy of monitoring tests of fellow eyes in patients with unilateral neovascular age-related macular degeneration: early detection of neovascular age-related macular degeneration study” (*Ophthalmology* 2021;128:1736-1747) would like to make the following correction (change in bold). The third author’s name should appear as follows: Augusto **Azuara**-Blanco.

