# Supplementary material for: Diagnostic Accuracy of Monitoring Tests of Fellow Eyes in Patients with Unilateral Neovascular Age-Related Macular Degeneration: Early Detection of Neovascular Age-Related Macular Degeneration Study
Source: Ophthalmology. 2021 Dec;128(12):1736–47. doi: 10.1016/j.ophtha.2021.07.025 (PMC8639888; doi:10.1016/j.ophtha.2021.07.025)
Supplement: Table S1 [file mmc1.pdf]

**Reference standard**	**Definition**	**Analyses**	**Outcome**	**N included**
Primary	Clinician determination of onset of active nAMD based on FFA	Any index test positive during the entire follow up period is counted as a test positive .	**Primary outcome**	120
		Any index test positive only from last 6 months of follow up counted as a test positive	Secondary outcome	
		Index test result from the last visit only	Secondary outcome	
Enhanced	Reading centre determination of onset of nAMD based on FFA	Index test results from the entire follow up period	Secondary outcome	118
Pragmatic	Clinician diagnosis of onset of active nAMD with or without FFA	Index test results from the entire follow up period	Secondary outcome	145
