# Supplementary material for: Diagnostic Accuracy of Monitoring Tests of Fellow Eyes in Patients with Unilateral Neovascular Age-Related Macular Degeneration: Early Detection of Neovascular Age-Related Macular Degeneration Study
Source: Ophthalmology. 2021 Dec;128(12):1736–47. doi: 10.1016/j.ophtha.2021.07.025 (PMC8639888; doi:10.1016/j.ophtha.2021.07.025)
Supplement: Table S2 [file mmc2.pdf]

Table S2– Number of visits/tests completed per participant over the follow up period

	Eligible participants (n=543)			
	Mean (SD), count	Minimum – maximum	Percentile 25 – Percentile 75	Percentile 95
Clinic visits (n 543)	15.6 (7.7)	1 - 35	10 - 21	28
Index tests				
Self-reported vision	14.0 (7.4)	0 - 35	9 - 19	27
*Amsler n = 453	13.8 (7.5)	0 - 35	8 - 18	26
Visual acuity	15.5 (7.6)	1 - 35	10 - 21	28
Fundus clinical examination	14.2 (7.4)	0 - 35	9 - 19	26
OCT	14.5 (7.5)	1 - 35	9 - 19	27

* excludes participants with a positive Amsler at baseline (n=90)
