# Supplementary material for: Diagnostic Accuracy of Monitoring Tests of Fellow Eyes in Patients with Unilateral Neovascular Age-Related Macular Degeneration: Early Detection of Neovascular Age-Related Macular Degeneration Study
Source: Ophthalmology. 2021 Dec;128(12):1736–47. doi: 10.1016/j.ophtha.2021.07.025 (PMC8639888; doi:10.1016/j.ophtha.2021.07.025)
Supplement: Table S3 [file mmc3.pdf]

Table S3 Interval in days between the primary reference standard (clinician determination of the presence or absence of active nAMD based on interpretation of a valid fluorescein angiogram) and index test, grouped by conversion to nAMD in EDNA study eye

	Participants who developed nAMD			Participants who did not develop nAMD		
	Number of participants	Mean no. days (SD)	Median P25 – P75	Number of participants	Mean no. days (SD)	Median P25 – P75
Self-reported vision	118	7.3 (13.4)	0, 0-13	337	8.4 (51.0)	0, 0-0
Amsler	98	30.3 (114.4)	0, 0-13	279	82.2 (223.0)	0, 0-0
Visual acuity	120	16.3 (78.2)	0, 0-13	335	117.0 (264.1)	0, 0-21
Fundus clinical examination	119	9.4 (28.5)	0, 0-13	335	9.7 (64.6)	0, 0-0
OCT	120	6.9 (17.2)	0, 0-12	335	39.0 (142.3)	0, 0-0

The number of participants differ by test as not all tests were performed at every visit. The Amsler was performed least number of times as 90 participants had a positive test at baseline which negated performance of this test as subsequent visits

P25 – P75 : percentile 25 – percentile 75
