# Supplementary material for: Diagnostic Accuracy of Monitoring Tests of Fellow Eyes in Patients with Unilateral Neovascular Age-Related Macular Degeneration: Early Detection of Neovascular Age-Related Macular Degeneration Study
Source: Ophthalmology. 2021 Dec;128(12):1736–47. doi: 10.1016/j.ophtha.2021.07.025 (PMC8639888; doi:10.1016/j.ophtha.2021.07.025)
Supplement: Table S4 [file mmc4.pdf]

Cross tabulations of FFA determination of conversion to nAMD by retina expert at clinical site (primary reference standard) versus independent reading center (enhanced reference standard)

| | | FFA interpretation by site clinician (primary reference standard) | | | |
|---|---|---|---|---|---|
| | | Positive | Negative | Inconclusive | Total |
| FFA interpretation at the reading center (enhanced reference standard) | Positive | 99 | 15 | 5 | 119 |
| | Negative | 20 | 318 | 3 | 341 |
| | Ungradable | 1 | 3 | 0 | 4 |
| | Total | 120 | 336 | 8 | 464 |
