# Supplementary material for: Diagnostic Accuracy of Monitoring Tests of Fellow Eyes in Patients with Unilateral Neovascular Age-Related Macular Degeneration: Early Detection of Neovascular Age-Related Macular Degeneration Study
Source: Ophthalmology. 2021 Dec;128(12):1736–47. doi: 10.1016/j.ophtha.2021.07.025 (PMC8639888; doi:10.1016/j.ophtha.2021.07.025)
Supplement: Table S5 [file mmc5.pdf]

	Pragmatic reference standard			
Index test	Sensitivity (%) (95% CI)	True positives / Participants with nAMD	Specificity (%) (95% CI)	True negatives / Participants without nAMD
Self-reported vision	3.5 (1.3, 8.1)	5 / 143	96.5 (94.0, 98.1)	334/346
Amsler test	33.7 (25.1, 43.5)	38 / 115	81.0 (76.0, 85.1	230/284
Visual acuity	31.7 (24.7, 39.7)	46 / 145	63.8 (58.6, 68.8)	219/343
Fundus clinical examination	54.5 (46.4, 62.5)	78/143	97.7 (95.4, 98.9)	335/343
OCT	92.4 (86.8, 95.8)	134 / 145	88.3 (84.5, 91.3)	303/343
