# Supplementary material for: Diagnostic Accuracy of Monitoring Tests of Fellow Eyes in Patients with Unilateral Neovascular Age-Related Macular Degeneration: Early Detection of Neovascular Age-Related Macular Degeneration Study
Source: Ophthalmology. 2021 Dec;128(12):1736–47. doi: 10.1016/j.ophtha.2021.07.025 (PMC8639888; doi:10.1016/j.ophtha.2021.07.025)
Supplement: Table S6 [file mmc6.pdf]

Table S6 Paired Comparison between tests

	Difference (%) in sensitivity (95% CI), p-value*	Difference (%) in specificity (95% CI), p-value*
OCT vs self-reported vision	87.4 (79.1 to 91.9), <0.001	-9.3 (-13.4 to -5.3), <0.001
OCT vs Amsler	59.6 (48.1 to 68.6), <0.001	6.9 (1.1 to 12.6), 0.02
OCT vs visual acuity	61.7 (49.9 to 70.7), <0.001	21.5 (15.6 to 27.3), <0.001
OCT vs fundus clinical examination	37.8 (27.6 to 47.1), <0.001	-9.9 (-13.9 to -6.1), <0.001
Amsler vs fundus clinical examination	-16.3 (-29.0 to -2.8), 0.03	-16.2 (-21.3 to -11.5), <0.001
Amsler vs self-reported vision	29.6 (19.2 to 39.7), <0.001	-17.3 (-22.3 to -12.7), <0.001
Amsler vs visual acuity	9.2 (-3.5 to 21.5), 0.21	12.3 (5.3 to 19.2), <0.001
Fundus clinical examination vs self-reported vision	50.0 (40.3 to 58.7), <0.001	0.6 (-2.0 to 3.3), 0.80
Fundus clinical examination vs visual acuity	24.4 (12.5 to 35.2), <0.001	31.3 (25.9 to 36.7), <0.001
Self-reported vision vs visual acuity	-25.2 (-34.0 to -16.5), <0.001	30.7 (25.7 to 35.9), <0.001

Paired comparisons show highly significant differences between OCT and other tests for sensitivity. For specificity significance is less between OCT and Amsler. There is no significant difference in sensitivity between OCT and Amsler or between fundus clinical examination and self reported vision for specificity.

*p-values are calculated using the McNemar test; differences are the first test minus the second.
