# Supplementary material for: Diagnostic Accuracy of Monitoring Tests of Fellow Eyes in Patients with Unilateral Neovascular Age-Related Macular Degeneration: Early Detection of Neovascular Age-Related Macular Degeneration Study
Source: Ophthalmology. 2021 Dec;128(12):1736–47. doi: 10.1016/j.ophtha.2021.07.025 (PMC8639888; doi:10.1016/j.ophtha.2021.07.025)
Supplement: Table S7 [file mmc7.pdf]

Supplementary Table S 7:

Likelihood positive and negative ratios and diagnostic odds ratios with 95% confidence intervals

	Likelihood positive ratio (95% CI)	Likelihood negative ratio (95% CI)	Diagnostic odds ratio (95% CI)
Self-reported vision	1.4 (0.6, 3.3)	1.0 (0.5, 1.8)	1.4 (<0.01, >100)
Amsler test	1.8 (1.3, 2.6)	0.8 (0.7, 1.0)	2.2 (<0.01, >100)
Visual acuity	0.9 (0.6, 1.2)	1.1 (1.0, 1.2)	0.8 (<0.01, >100)
Fundus clinical examination	22.4 (16.7, 30.1)	0.5 (0.2, 0.9)	47.4 (<0.01, >100)
OCT	7.5 (4.1, 13.9)	0.1 (0.1, 0.1)	79.5 (<0.01, >100)
