# Supplementary material for: Diagnostic Accuracy of Monitoring Tests of Fellow Eyes in Patients with Unilateral Neovascular Age-Related Macular Degeneration: Early Detection of Neovascular Age-Related Macular Degeneration Study
Source: Ophthalmology. 2021 Dec;128(12):1736–47. doi: 10.1016/j.ophtha.2021.07.025 (PMC8639888; doi:10.1016/j.ophtha.2021.07.025)
Supplement: Table S8 [file mmc8.pdf]

Table S8 Sensitivity and specificity for combinations of index tests when either test is positive.

**OCT combined with**	**Sensitivity (%) (95% CI)**	**True positives / Participants with nAMD**	**Specificity (%) (95% CI)**	**True negatives / Participants without nAMD**
Self-reported vision	91.5 (84.9, 95.5)	108 / 118	85.1 (80.8, 88.5)	285 / 335
Amsler	93.9 (87.0, 97.4)	92 / 98	73.3 (67.8, 78.2)	203 / 277
Visual acuity	96.7 (91.5, 99.0)	116 / 120	60.0 (54.7, 65.1)	201 / 335
Fundus clinical examination	94.1 (88.1, 97.3)	112 / 119	86.0 (81.8, 89.3)	288 / 335

Sensitivity and specificity with 95% confidence interval (CI) of combinations of OCT when either test is positive and this definition specifies that both tests have to be negative for the combination to be deemed negative. The combinations show a marginal improvement in sensitivity compared to OCT alone Specificities are lower compared to OCT alone.
