# Supplementary material for: Diagnostic Accuracy of Monitoring Tests of Fellow Eyes in Patients with Unilateral Neovascular Age-Related Macular Degeneration: Early Detection of Neovascular Age-Related Macular Degeneration Study
Source: Ophthalmology. 2021 Dec;128(12):1736–47. doi: 10.1016/j.ophtha.2021.07.025 (PMC8639888; doi:10.1016/j.ophtha.2021.07.025)
Supplement: Table S9 [file mmc9.pdf]

**Table S9** Sensitivity and specificity of combinations of index test if both tests are positive

| OCT results combined with* | Sensitivity (%) (95% CI) | True positives / Participants with nAMD | Specificity (%) (95% CI) | True negatives / Participants without nAMD |
|---|---|---|---|---|
| Self-reported vision | 4.2<br>(1.6, 9.8) | 5 / 118 | 99.7<br>(98.2, 100.0) | 334 / 335 |
| Amsler | 32.7<br>(24.2, 42.5) | 32 / 98 | 96.8<br>(93.9, 98.4) | 268 / 277 |
| Visual acuity | 25.0<br>(18.1, 33.5) | 30 / 120 | 94.0<br>(90.9, 96.1) | 315 / 335 |
| Fundus clinical examination | 51.3<br>(42.4, 60.1) | 61 / 119 | 99.4<br>(97.7, 100.0) | 333 / 335 |

Sensitivity and specificity with 95% confidence intervals (CI )of combinations of OCT with the other index tests when the definition is positive only if **both** OCT **and** the index test results are positive. Sensitivity drops compared to the definition of either positive but specificity improves for all combinations tested.
