# Supplementary material for: Diagnostic Accuracy of Monitoring Tests of Fellow Eyes in Patients with Unilateral Neovascular Age-Related Macular Degeneration: Early Detection of Neovascular Age-Related Macular Degeneration Study
Source: Ophthalmology. 2021 Dec;128(12):1736–47. doi: 10.1016/j.ophtha.2021.07.025 (PMC8639888; doi:10.1016/j.ophtha.2021.07.025)
Supplement: Table S10 [file mmc10.pdf]

Table S10

Combination is positive if any included test is positive	Sensitivity (%) (95% CI)	True positives / Participants with nAMD	Specificity (%) (95% CI)	True negatives / Participants without nAMD
Fundus examination OR Self-reported vision OR Amsler OR VA	72.2 (62.5, 80.1)	70 / 97	55.6 (49.7, 61.3)	154 / 277
Self-reported vision OR Amsler OR VA	50.0 (40.3, 59.7)	49 / 98	57.0 (51.2, 62.7)	158 / 277

Sensitivity and specificity of combinations of index tests after exclusion of OCT (upper row) and after exclusion of both OCT and fundus clinical examination (lower row). The definition is positive if any of the tests in the combination is positive. Sensitivity is reduced by the exclusion of both fundus examination and OCT while specificity is only marginally altered.
