# Supplementary material for: Diagnostic Accuracy of Monitoring Tests of Fellow Eyes in Patients with Unilateral Neovascular Age-Related Macular Degeneration: Early Detection of Neovascular Age-Related Macular Degeneration Study
Source: Ophthalmology. 2021 Dec;128(12):1736–47. doi: 10.1016/j.ophtha.2021.07.025 (PMC8639888; doi:10.1016/j.ophtha.2021.07.025)
Supplement: Appendix S1 [file mmc11.pdf]

Supplementary material Appendix 1 (Appendix S1)

Assessments performed at the baseline study visit and follow up.

- **Amsler test**

The patient should complete the Amsler within the EDNA case report form (CRF) for the EDNA study eye according to the instructions on the CRF clearly explained to them. Make sure the patient is aware of the eye to be occluded. Please emphasize that the eye to be tested is the EDNA study eye and the eye to be occluded is the fellow eye which is on active treatment for nAMD.

After completion, record on the baseline CRF whether there are any distortion or scotoma marks on the Amsler grid. If the patient has a baseline distortion or scotoma they are still eligible to take part in EDNA. However, no further Amsler tests need to be collected as part of EDNA follow-up.

- **Visual acuity**

Visual acuity should be measured using a LogMAR/EDTRS chart calibrated for the distance used and recorded in number of letters read for both eyes. A logMAR/EDTRS conversion chart is within the CRF. Whether the BCVA is undertaken using pinhole/refraction/unaided/using habitual refraction should also be recorded. N.B. The same method should be used for all follow-up appointments so if follow-up visions are routinely performed using a different method to baseline please record baseline VA using the routine follow-up method.

- **OCT findings in study eye**

No validation is required to conduct the OCT for the EDNA study. Please follow the CARF protocol for image acquisition. If an enhanced depth imaging (EDI) scan of the choroid is collected this can be submitted as an optional extra. Any study team member who can interpret tomograms can complete this section of the CRF. The patient is ineligible if baseline OCT shows signs of late AMD. Section 3.2.5 of the EDNA protocol gives further details of possible abnormalities which may be detected by clinical interpretation of the OCT.

- **Fundus examination of study eye**

This can be either assessed by a Slit Lamp clinical examination or from fundus photography. We recommend that colour fundus photography includes fields 1 and 2 comprising of the central macula. If a wide field camera is used a single image is sufficient. The examination of the eye and or the colour fundus images with completion of the CRF can be made by any appropriately qualified member of the study team.

No AMD No drusen or pigmentary irregularities or only small hard drusen

Early AMD Soft drusen and/or pigmentary irregularities

Late AMD Exudative Neovascular AMD or Geographic atrophy: At baseline if Geographic atrophy with sufficient vision (VA≥68) patient is eligible and if nAMD detected at baseline the patient is ineligible.

- **FFA**

A fluorescein angiogram conducted on referral should confirm that the patient has no signs of nAMD in the EDNA study eye. If an FFA has already been conducted, there is no need to repeat an FFA for EDNA but the results of baseline FFA should be checked to ensure the patient is eligible at baseline. The FFA can be undertaken on any image acquisition system using local site protocols for its conduct.

Data collection instructions to participants and study team on study procedures

Study Number | | | | | |

Date D D / M M / Y Y Y Y

How is your vision in the untreated eye, compared with the last visit?
Please see below four possible answers. We would ask you to tick one box (only)

About the same or better	A bit worse	Worse	Much worse

NB: Amsler Tests are to be completed on the <u>EDNA study eye ONLY</u>

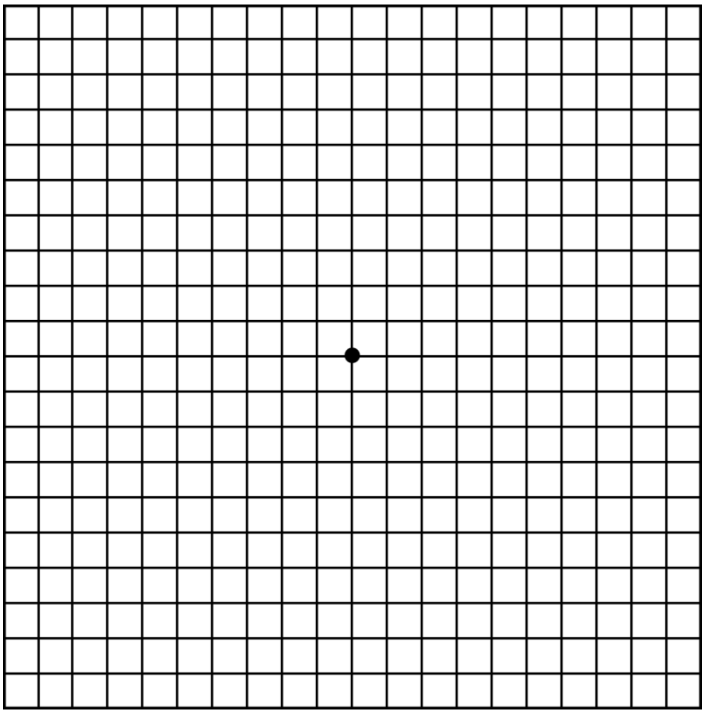

Instructions for Use

1. **Wear the glasses you would normally use when reading.**
2. **Hold the chart at the distance at which you would read a book. Cover the eye in which you are getting injections so that you are looking at the chart with your EDNA study eye.**
3. **Fix your gaze on the centre black dot**
4. **Keeping your gaze fixed on the black dot, try to see if any lines are distorted or missing.**
5. **If you notice that the lines are not straight or blurred please mark the region of the chart that is abnormal with a pen or pencil; If the lines on the chart look normal tick this box** ☐
6. ***Always* keep the Amsler's Chart the *same distance* from your eyes each time you test.**

1. VISUAL ACUITY

No of letters RE:________ No of letters LE: _______

Has visual acuity reduced by 10 letters or more in STUDY EYE since baseline study visit?

Yes ☐ No ☐ Not done ☐ If not done give reason ______________________________

2. OCT (STUDY EYE)

OCT's are captured on both eyes at every visit according to local site protocol for acquisition. Site study staff recorded whether the OCT showed signs of neovascular AMD on a structured data capture form

Does the OCT in STUDY EYE show signs of neovascular AMD?

SRF on OCT Yes ☐ No ☐ Neovascular PED on OCT Yes ☐ No ☐

IRF on OCT Yes ☐ No ☐ Any other reason for OCT being positive? Yes ☐ No ☐

If yes please give reason ____

OCT not done ☐ If not done give reason ______________________________

3. FUNDUS EVALUATION (STUDY EYE)

Method of assessment:

Colour fundus photography? Yes ☐ No ☐ Slit lamp biomicroscopy? Yes ☐ No ☐

Clinical signs of nAMD on Fundus? Yes ☐ No ☐ Not done ☐

If not done give reason ____

4. FFA acquisition and EVALUATION (STUDY EYE) by clinican

FFA's are performed in clinical sites according to local protocol. These FFA's evaluated by the site clinicians for the presence of signs of neovascular AMD. The detection of regions of hyperfluoresence during the run of the FFA which intensifies and expands is taken as a sign of extravasation from abnormal leaky blood vessels in the macular area of the fundus. Clinicians use prior knowledge of hyperfluorescence patterns and temporal associations to distinguish similar appearances that can arise from atrophy and or other macular pathology. Clinicans record if in their opinion exudative AMD was present.

5. FFA EVALUATION (STUDY EYE) by reading centre

FFA evaluation in the reading centre. Trained graders reviewed the angiograms without access to the other index tests and graded for presence of nAMD in the EDNA study eye. Anonymised FA images were displayed on screens and images graded according to the protocol outlined below.

Is Exudative AMD Present:

**Grade Options**	
Yes	If Yes:

No	Continue to Is CNV or RAP active question
CG	

The second part covers whether there is an active CNV or RAP present, or if the lesion is inactive, such as fibrosis with no leakage of fluorescein dye in the late FFA images, just staining of the lesion.
Is CNV or RAP Active:

**Grade Options**	
Yes	If Yes:
No	Continue with the lesion component questions

1.2 Angiographic Features of Lesion Components

This section of the protocol will provide the definitions for all lesion components to be graded on FA and a description of how they should be graded.

1.2.1 Lesion Components

This refers to any of the features of exudative AMD for which measurements must be recorded. These components are:

• Classic CNV
• Occult CNV (FPED/LLIO)
• RAP
• Blocked Fluorescence
• RPE tear
• SPED
• Fibrosis
• Atrophy within the lesion

Total lesion is defined as the total area of all of the components mentioned above.

1.2.2 Choroidal Neovascularisation (CNV)

Choroidal Neovascularisation (CNV) is an in-growth of choroidal capillaries through a break in the outer aspect of Bruch's membrane. CNV is easily visible in FA images, unlike in colour images.

Delineating the boundaries of CNV in eyes with AMD can be quite difficult as it is often accompanied by in-growth of fibrous scar tissue, and may have a variety of complex angiographic appearances.

Traditionally, CNV has been divided into two categories; classic and occult. These can only be determined in FA images.

In this protocol, CNV is described based on when it typically appears on the FFA run.

1 - Area of early bright hyperfluorescence: Classic and RAP
2 - Area of mid-late hyperfluorescence: FPED and LLIO

Lesion types with an area of early bright hyperfluorescence

This category will contain classic CNV and RAP lesions.

1.2.2.1 Classic CNV

Classic CNV is typically recognised by the appearance of a well-demarcated area or areas of hyperfluorescence which occur in the early phase of the angiographic sequence. The hyperfluorescence represents the presence of fluorescein dye in the abnormal vascular complexes. Initially, restricted by the

walls of the vasculature, the hyperfluorescent dye may take on the form of vascular profiles (lacy network) or may appear as areas of homogenous but well delineated hyperfluorescence. In later phases of the angiogram, progressive pooling of fluorescein dye which has leaked out of the CNV into the overlying sub-sensory retinal space usually obscures the boundaries of the initially well demarcated region of hyperfluorescence. In the slow-filling form of classic CNV, the vessels should be discernible in the early phase of the angiogram, but may not be well visualised until about 2 minutes after dye injection, with late-phase leakage into the overlying sub-retinal space 5 and 10 minutes after injection. A flat rim of blocked fluorescence is also common. 'Older' classic lesions also tend to be more blocked in the centre, showing more activity at their borders than in the central region; it is therefore possible that although there is early hyperfluorescence, the centre of the classic lesion may hypofluoresce, and smooth leakage may also be present.

- Classic CNV should be measured during the early fill phase, before leakage breaks the boundaries of the classic lesion. Images selected before 30 seconds (20-30 seconds) are recommended as this is the earliest time that everything is filled, yet not all the classic CNV indents have gone. (By 40 seconds the indented edge observed at 25-30 seconds has disappeared and the leakage is also brighter and passes its own margins. CNV should therefore be measured after the new vessels are filled, but before leakage begins).
- If there are two or more classic lesions present, quite often there is something connecting them (feeder vessel). All areas of classic CNV should be measured and added together.
- Areas that might be considered to have little or no leakage and are surrounded by classic CNV should be included as part of the classic CNV, providing there is an arc of at least 270 degrees of classic CNV surrounding the area of non-leakage.
- Slow-filling classic can sometimes be confused with FPED. If a grader is unsure if a lesion is slow-filling classic or occult, it is advised that the grader observe how the lesion changes over time (at 30 seconds, 1 minute, 5 minutes and 10 minutes). If there is leakage and it does not look Occult, it can be graded as Classic. The grader needs to check if RPE is flat or elevated on stereo images, this is best seen at around 2-3 mins before profuse leakage really starts, to determine if FPED is present or absent.

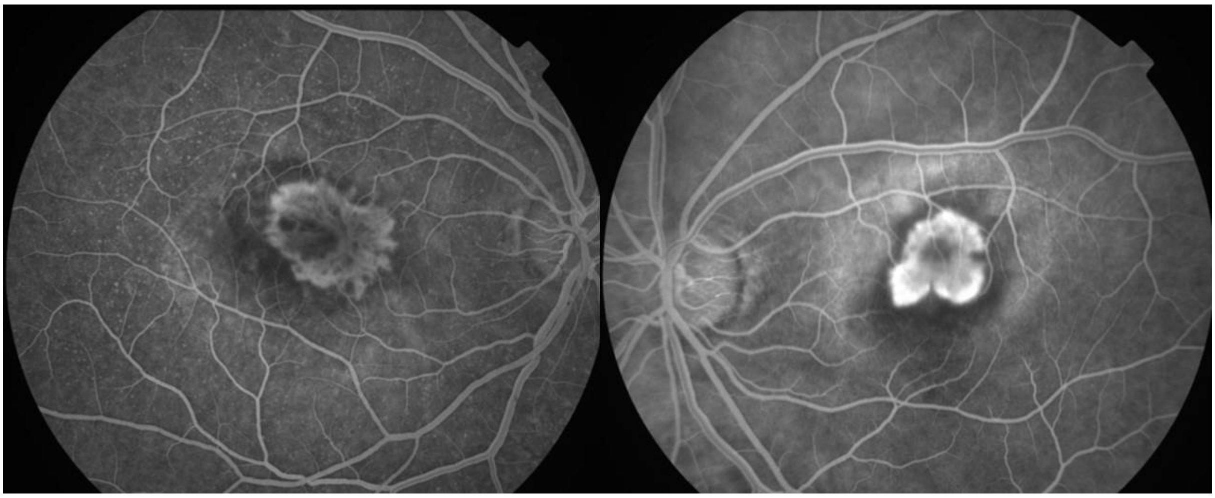

Fig 1. Classic CNV

**Grade Options**			
Yes	If Yes:		
No	Area	*measurement of area/s*	mm²
	Location	Sub-foveal / Juxta-foveal /Extra-foveal	

1.2.2.2 Retinal Angiomatous Proliferation (RAP)

Angiomatous proliferations originate from the retina and extend posteriorly into the sub-retinal space, eventually communicating in some cases with choroidal new vessels. RAPs are considered to be a distinct sub-group of neovascular AMD. In CNV, venules erode through the RPE and infiltrate the sub-RPE and sub-retinal spaces. CNV also communicates with the retinal circulation (referred to as a retinal-choroidal anastomosis (RCA). RAPs on the other hand, begin as fronds of intra-retinal neovascularisation, (Yannuzzi Stage 1). These fronds may grow into the sub-retinal space causing serous retinal pigment epithelial

detachments (SPEDs) (Yannuzzi Stage 2) and ultimately anastomose with choroidal neovascular complexes (Yannuzzi Stage 3). RAP can be accompanied by drusen, exudate and lipid.

[See appendix A for Yannuzzi descriptions and sample drawings]

When looking for a RAP vessel, it is important to look at a very early stereo pair of the transit run, ideally up to 30 secs, and appreciate an elevated perifoveal retinal vessel which doesn't taper, but instead does a very sharp right angled turn usually downwards towards the RPE.

This vascular complex then goes on to leak profusely in the later images. This needs to be present and identified before we can confidently diagnose RAP, however other secondary clues can also be helpful, such as superficial intra-retinal haemorrhages, exudates and SRF/SPED/FPED etc. Appearance of a retinal-retinal anastomosis is also a strong indicator of RAP.

RAP should be measured on an early frame before there is evidence of leakage. If more than one RAP lesion is present, all RAP lesions should be measured and the areas summed for RAP area.

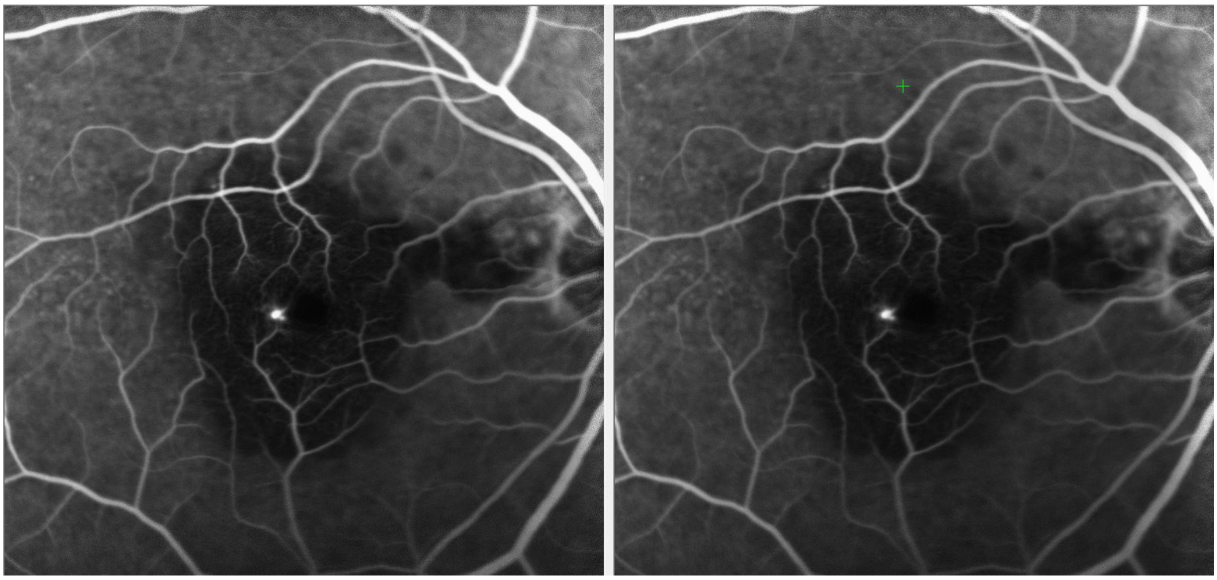

Fig 2. RAP. Early FFA (stereo), note small hyperfluorescent spot centrally

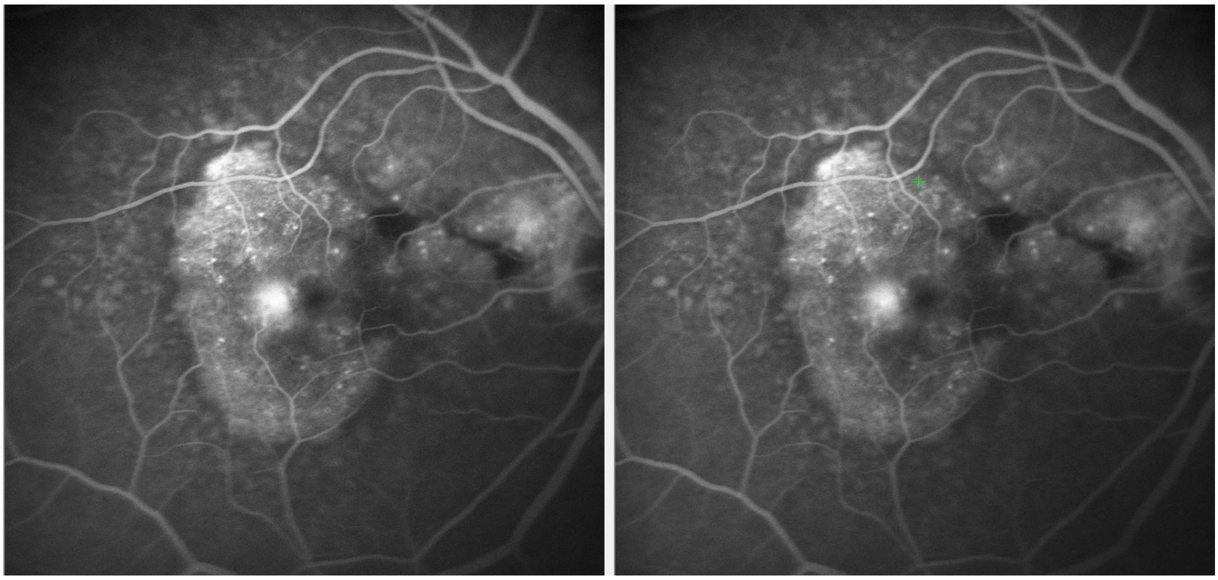

Fig 3. RAP. Late FFA (stereo)

Extra Notes on RAP Measurement

When drawing an FPED that has multiple RAPs within its area, then the sum of the areas of RAP should be subtracted from the area of FPED. For example, if the total area of FPED is 2mm² (measured around the

boundary of FPED), and the area of multiple RAP within is 0.5mm², then the area of FPED should be recorded as 1.5mm² and the area of RAP as 0.5mm².

**Grade Options**			
Yes	If Yes:		
No	Area	*measurement of area/s*	mm²
	Location	Sub-foveal / Juxta-foveal /Extra-foveal	

Lesion types with an area of mid-late hyperfluorescence

This category will contain both occult CNV types; FPED and LLIO. If the grader identifies PFED or LLIO, this should be graded under the Occult CNV lesion component.

The appearance of occult CNV varies widely, and it may be difficult to define the extent or to be confident of its presence. Occult patterns usually only start to show from approximately the one-minute images. Typically, occult CNV lesions leak less than classic CNV.
Occult CNV is generally speckled and may show only fluorescein staining.

The identification of occult CNV in which irregular elevation of the RPE is usually present, is facilitated by stereoscopic viewing of the images, this is best viewed at around 2-3 min. There are two forms of hyperfluorescence which are identified as 'occult' forms of CNV: fibrovascular pigment epithelial detachment (FPED) & late leakage of indeterminate origin (LLIO).

[Graders should take care when they observe such stippled hyperfluorescence as it may be transmission defect. To check, graders should refer to the colour images and check for signs of RPE hypo-pigmentation. RPE atrophy can look like occult CNV. It is also important not to confuse stippled hyperfluorescence with drusen. Hard drusen fluoresce brightly in the early phase of the angiogram and then slowly fade. Soft drusen fluoresce more slowly and remain fluorescent for longer.]

1.2.2.3 Fibrovascular Pigment Epithelial Detachment (FPED)

This is a type of occult neovascularisation in which areas of usually irregularly or undulating elevated stippled hyperfluorescence are seen on stereoscopic angiography within 1 to 2 minutes after fluorescein injection (early or mid-phases). These areas are neither as discrete nor as bright as areas of classic CNV in the transit phase of the angiogram. By 10 minutes after injection, there is persistent fluorescein staining or leakage within the sub-sensory retinal space overlying this detachment.

Both the contours of the elevation and the border of the elevation (at its base) tend to be irregular. The borders of elevated RPE often slope gradually downward to surrounding flat RPE so that the demarcation between elevated RPE and flat RPE cannot be easily determined.

Frequently, the intensity of fluorescence at the boundary of the elevated RPE is quite irregular, with some areas fading relative to the fluorescence of the remaining areas of elevated RPE (making it difficult to distinguish the boundaries of fading occult CNV fluorescence from the fading fluorescence of the surrounding RPE).

Stipples of hyperfluorescence are often intermixed on the surface of the elevated staining/leaking RPE. These hyperfluorescent areas are neither as discrete nor as bright as areas of classic CNV or Serous PEDs (SPED) in the transit (early) phase of the angiogram.

If the surface of the FPED is smooth, it can usually be differentiated from a SPED by the irregular contour of its base, and the uneven pattern of hyperfluorescence. Since the elevation of the FPED tends to be lower than it is in SPEDs, and in many cases is quite subtle, good stereo effect is essential in determining their presence. Graders should view several stereo pairs to achieve the optimum stereoscopic effect for drawing the FPED.

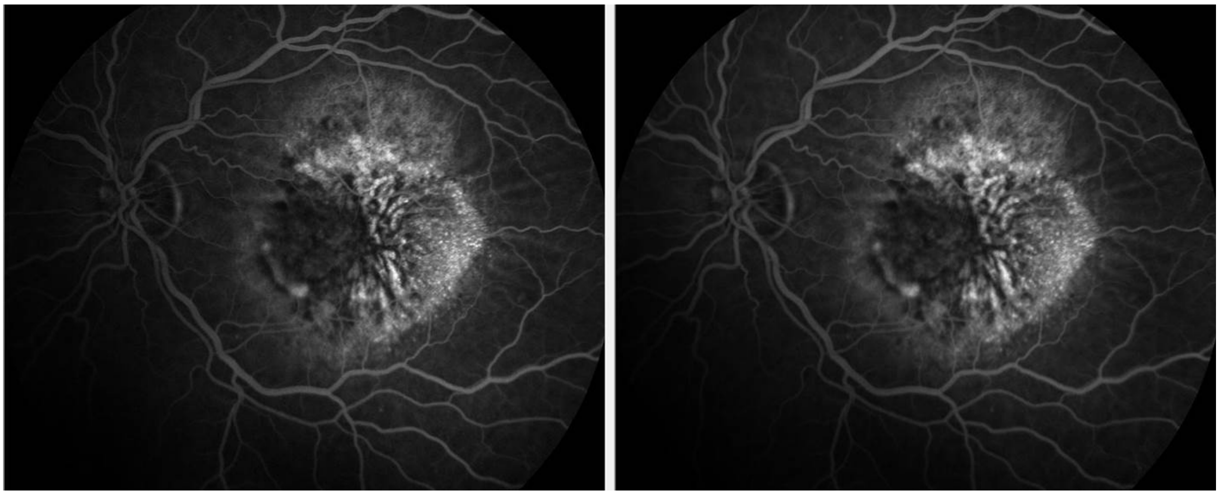

Fig 4. FVPED (Stereo)

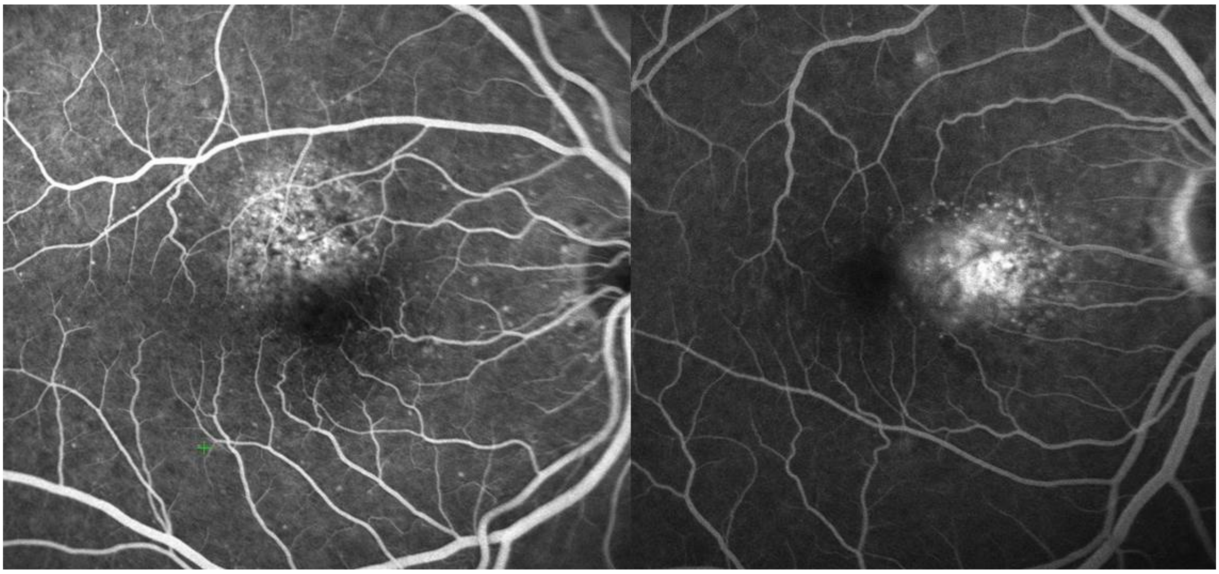

Fig 5. FVPED (note the stippled appearance)

**Grade Options**			
Yes	If Yes:		
No	Area	*measurement of area/s*	mm^2
	Location	Sub-foveal / Juxta-foveal /Extra-foveal	

1.2.2.4 Late-Phase Leakage of Indeterminate Origin (LLIO)

This is a type of occult CNV in which poorly demarcated areas of leakage appear at the level of the RPE in the later phases of the angiogram (2-5 minutes) without well-demarcated areas of hyperfluorescence discernible in the early phase of the angiogram that account for fluorescein leakage.

This type of leakage is seen as hyperfluorescence, which is brighter and deeper than the hyperfluorescence associated with fluorescein dye that has leaked in the sub-retinal space. Some useful cues are the proximity of the hyperfluorescence to the RPE. Overlying SRF is usually elevated on stereoscopic examination when compared with adjacent healthy RPE. Stereo viewing will aid in the identification of late ill-defined leakage of indeterminate origin.

[Graders should distinguish LLIO in occult CNV from slow filling classic CNV. In classic CNV with slow filling of choroidal vessels, vessels are discernible in the early phase of the angiogram.]

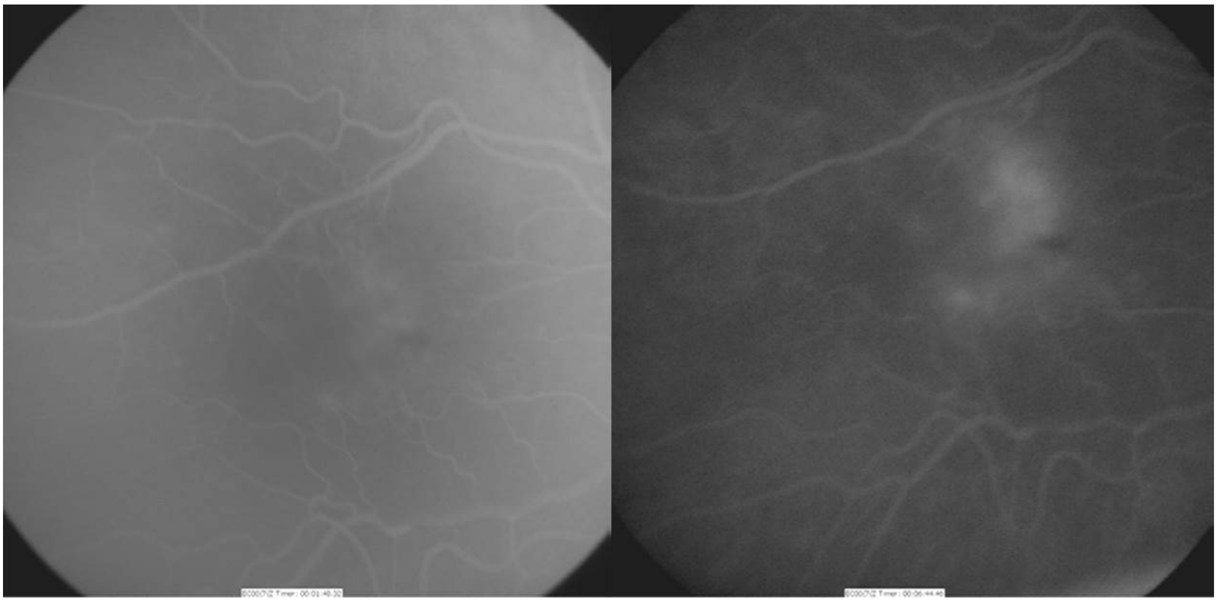

Fig 6. Occult CNV – LLIO

Occult CNV (FPED) should be observed on an image at approximately 1 minute. Occult CNV (LLIO) should be observed at the latest time possible (approximately 10-minute frame). Graders should then mentally transfer these observations to the image selected for drawing. Drawings should be made on a suitable image, preferably before 1 minute. If frames after approximately 7 minutes are not available, the grader should mark this in the image quality section.

As with classic CNV, occult CNV should be measured after the CNV is filled, but before leakage begins.
