# Supplementary material for: Diagnostic Accuracy of Monitoring Tests of Fellow Eyes in Patients with Unilateral Neovascular Age-Related Macular Degeneration: Early Detection of Neovascular Age-Related Macular Degeneration Study
Source: Ophthalmology. 2021 Dec;128(12):1736–47. doi: 10.1016/j.ophtha.2021.07.025 (PMC8639888; doi:10.1016/j.ophtha.2021.07.025)
Supplement: Figure S1 [file mmc12.pdf]

EDNA STUDY DESIGN AND PROCEDURES

Individuals age 50 and over with newly diagnosed nAMD

Eligibility Criteria

- Newly diagnosed neovascular AMD in one eye only with diagnosis confirmed by FFA
- Unaffected eye (EDNA study eye) confirmed by FA and OCT to be free of nAMD
- About to commence or recently commenced anti VEGF therapy in affected eye
- Age 50-95

<u>Exclusion Criteria</u>

- unwilling to participate or unable to give informed consent
- history of nAMD in both eyes
- nAMD in study eye detected at baseline
- presenting VA worse than 68 letters
- retinal pathology in study eye which can confound subsequent assessments
- not undergoing regular monitoring in standard of care
- FFA contraindicated
- patients whose baseline FFA

Individuals eligible and interested in participating, undergoing monitoring tests performed as standard care: VA, fundus examination/colour photography, OCT and FFA

Baseline study visit within 6 weeks of diagnostic FFA

- Confirmation of eligibility criteria fulfilled
- Consent obtained
- Training using Amsler test
- Baseline measures collected. The diagnostic tests performed as SOC may be used but any tests not already performed can be undertaken at the EDNA baseline study visit
- Blood collection (optional consent)

Clinic monitoring visit (standard of care) -

Collection of EDNA diagnostic tests [*Test positive definition*]

- Visual acuity [*reduction in VA from baseline≥10 letters*]
- OCT [*signs of fluid on OCT scan*]
- Amsler test [*appearance of new distortion /blank spots when none previously or clear evidence of increase in area of distortion/scotoma*]
- Fundus evaluation [*signs of nAMD on fundus*]
- Patient's subjective assessment of vision [*"much worse" vision*]

Where used at the local site OCTA may also be collected

Any test positive → FFA within one month

(+ OCTA data collection if available at local site)

All tests negative
(or indeterminate)

Following FFA if clinical diagnosis of nAMD in the study eye →
- refer to service clinic
- blood collection (optional consent)
- no further study visits required
- post exit monitoring (casenote review)

Following FFA if clinical diagnosis is no nAMD in the study eye

Continue monitoring as per local practice (FFA at 18 and exit appointment)

Loss to follow-up
