# Supplementary material for: Diagnostic Accuracy of Monitoring Tests of Fellow Eyes in Patients with Unilateral Neovascular Age-Related Macular Degeneration: Early Detection of Neovascular Age-Related Macular Degeneration Study
Source: Ophthalmology. 2021 Dec;128(12):1736–47. doi: 10.1016/j.ophtha.2021.07.025 (PMC8639888; doi:10.1016/j.ophtha.2021.07.025)
Supplement: Figure S2 [file mmc13.pdf]

Figure S 2. KM survival graph of time to onset of nAMD in the EDNA study eye

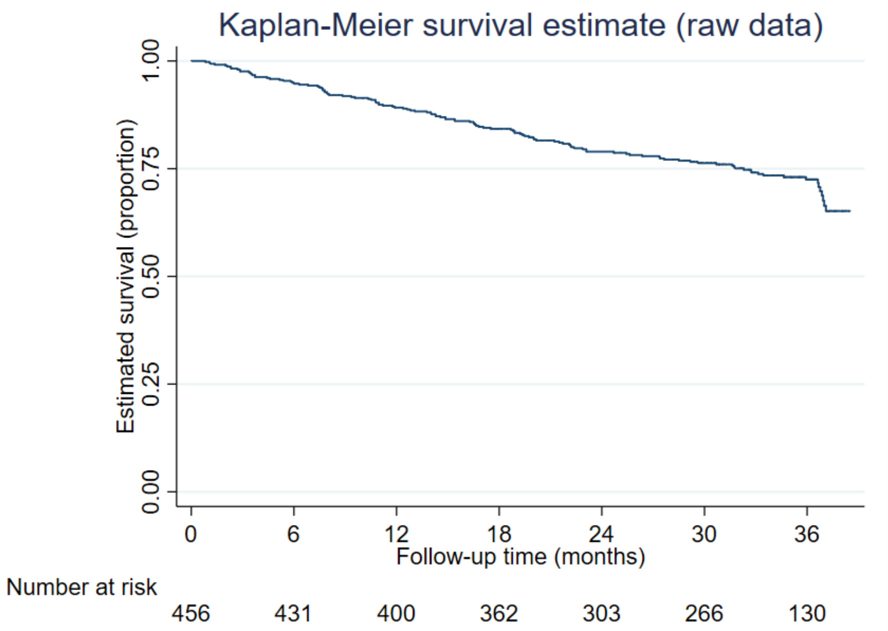

Kaplan Meier survival curve. Participants were censored on the date on which a positive determination of conversion to nAMD was made by the site clinician on the basis of an FFA. The X axis represents months from consent until development of nAMD or exit from the study. The crude conversion rate based the primary reference standard is 26% (95% CI 22.3, 30.6)
